# The Child under School Age

**Published:** 1912-10-12

**Authors:** 


### The Hospital

The Workers' Newspaper of Administrative Medicine and Institutional Life, Administration, National Insurance and Health.

No. 1371, Vol. LIII. SATURDAY, OCTOBER 12, 1912. PRICE ONE PENNY.

### THE CHILD UNDER SCHOOL AGE.

Medical inspection of school-going children has already yielded invaluable results, and has now come to be looked upon as indispensable in a community that has at heart the interests of the growing population. It would be a work of supererogation to argue in its favour, since the principles on which it is based are nowadays so generally admitted. But the logical conclusion of the premiss that enunciates these principles is not yet so popular as it ought to be. It is, of course, that if it is necessary medically to inspect school children, and to treat the lesions discovered by such inspection, it is much more desirable and much more necessary to inspect and treat children who are too young yet to attend school. Efficient supervision and prophylaxis before the child is forced to attend school are obviously good things: to expend money in securing them would, as obviously, be an economical policy, for it would tend to obviate future medical treatment and thereby effect a saving of time, trouble, and energy. There are vast numbers of children below school-going age who are never medically inspected and of whose condition and circumstances we know little, just as before the time of organised school medical inspection we knew little concerning the physical status of the school-going child. How are these out-of-school children to be reached? That some attempt must be made to deal with the problem of infant hygiene, if we are to preserve the fit and help those who cannot always efficiently help themselves, is self-evident. The question is merely in what manner is it possible to glean definite knowledge regarding certain points on which adequate information is necessary before we can delineate a policy in consonance with the best modern teaching.

This question has of late years aroused considerable interest. Only recently at a public meeting held in London the National Association for the Prevention of Infant Mortality and the Promotion of the Welfare of Children Under School Age was formally called into being. This Association is under Royal patronage, and should do excellent national service in the future, for the problems with which it is concerned are of national and imperial importance. The President of the Local Government Board, in presiding at the third public Conference on Infant Mortality, recognised the value both of the Conference and of the Association, and declared that their "influence, stimulus, and achievements have been general and widespread," a testimonial which will be heartily endorsed by everyone who has followed the work that has been done in relation to the problems of infant mortality during the past six years. The figures from the Foundling Hospital statistics, quoted by Mr. Burns, show very clearly how magnificent has been the work of prevention and amelioration that has been the main feature of philanthropic propaganda in this connection. Yet it is evident that a great deal has yet to be done before the public at large realises the imminence of the dangers that threaten the nation if important and urgent questions of health are neglected. Mr. Burns has lauded the energy of the professional press, but it is not the professional press that will evoke an enthusiastic campaign and call out the best energies of the laymen. Doctors, nurses, and district visitors know the truth: it is the laymen and laywomen who need enlightenment such as can and must be given in the lay press. That work of educating the public and of making known the dangers and their preventatives and remedies must fall on the new Association, and it is hardly necessary to say that the best wishes and the practical help and sympathy of the profession are at its service. What the Association must do, primarily, is to inculcate upon the public the fact that it is the period before school-going age that definitely matters in the formation of character, health, and mental and moral stamina of the average child. Mr. Burns is wholly right when he proclaims this fact in the somewhat epigrammatic sentence: "Let me decide the food, the home, and the conditions of life of every child, from birth to seven years of age, and the rest of mankind can do with the children after seven years of age what they like.''

The Association, it is satisfactory to note, has decided to call an English-speaking Conference to deal with the whole problem of the child under school age. A provisional committee, consisting of such recognised pioneers as Alderman Broadbent, Dr. James Kerr, and Sir Lauder Brunton, has been formed to make preliminary arrangements. It is proposed that the Conference shall take place in London on the occasion of the meeting of the International Medical Congress next year, and we are informed that the response to the invitations extended to the English-speaking world has been highly gratifying as showing the general interest which is taken in the problem. Similar conferences, international in character and polyglottic in speech, have been held from time to time, but their deliberations have lacked the national character which it is proposed to give to next year's meeting, and the difficulties English delegates have experienced in following discussions in French or German have made it advisable to limit the debates to English. This, while it may detract something from the general interest of the Conference, will certainly enhance its particular interest, for it will enable the problem to be attacked from a side which specially appeals to Englishmen and Englishwomen. The Association is to be congratulated on the decision to hold the Conference at a time when the International Congress will attract to London many of the foremost scientific exponents of the modern methods of safeguarding the infant population from the effects of environmental conditions, and it is to be hoped that the meetings will be thoroughly practical, and lead, finally, to a scheme for dealing with the problems in a satisfactory manner.

